# Impact of telehealth on general practice contacts: findings from the whole systems demonstrator cluster randomised trial

**DOI:** 10.1186/1472-6963-13-395

**Published:** 2013-10-08

**Authors:** Martin Bardsley, Adam Steventon, Helen Doll

**Affiliations:** 1 The Nuffield Trust, 59 New Cavendish Street, W1G 7LP London, UK; 2 Norwich Medical School, University of East Anglia, Chancellors Drive, NR4 7TJ Norwich, UK; 3 Oxford Outcomes, Seacourt Tower, West Way, OX2 0JJ Oxford, UK

**Keywords:** Telemedicine, Telemonitoring, General practice, Workload, Chronic disease

## Abstract

**Background:**

Telehealth is increasingly used in the care of people with long term conditions. Whilst many studies look at the impacts of the technology on hospital use, few look at how it changes contacts with primary care professionals. The aim of this paper was to assess the impacts of home-based telehealth interventions on general practice contacts.

**Method:**

Secondary analysis of data from a Department of Health funded cluster-randomised trial with 179 general practices in three areas of England randomly assigned to offer telehealth or usual care to eligible patients. Telehealth included remote exchange of vitals signs and symptoms data between patients and healthcare professionals as part of the continuing management of patients. Usual care reflected the range of services otherwise available in the sites, excluding telehealth. Anonymised data from GP systems were used to construct person level histories for control and intervention patients. We tested for differences in numbers of general practitioner and practice nurse contacts over twelve months and in the number of clinical readings recorded on general practice systems over twelve months.

**Results:**

3,230 people with diabetes, chronic obstructive pulmonary disease or heart failure were recruited in 2008 and 2009. 1219 intervention and 1098 control cases were available for analysis. No statistically significant differences were detected in the numbers of general practitioner or practice nurse contacts between intervention and control groups during the trial, or in the numbers of clinical readings recorded on the general practice systems.

**Conclusions:**

Telehealth did not appear associated with different levels of contact with general practitioners and practice nurses. We note that the way that telehealth impacts on primary care roles may be influenced by a number of other features in the health system. The challenge is to ensure that these systems lead to better integration of care than fragmentation.

**Trial registration number:**

International Standard Randomised Controlled Trial Number Register ISRCTN43002091.

## Background

The use of telehealth to allow patients with long-term health conditions to monitor vital signs and transfer readings to health professionals working remotely is increasingly being advocated as a way of delivering higher quality care more efficiently for better management of people with long term conditions [1]. Very often, one of the benefits of telehealth is perceived to be its potential impact on the use of hospital care. Relatively little attention has been placed on its impacts on primary care services such as general practices. Yet these are important determinants of patient experience and quality of care and the costs of providing primary and community health care to populations with long-term health conditions can be almost as large as hospital costs [2]. The impact of these technologies on primary care is therefore an important element in understanding the opportunities and potential barriers to introducing telehealth [3].

Two alternative hypotheses exist for the potential impact of telehealth on primary care. First, telehealth might reduce the need for support from primary care; for example, because problems are detected earlier, patients develop better self-care skills, or there is less need to undertake measurements such as weight in general practice. Telehealth may also serve to buffer the general practice in situations where the professionals who are monitoring patients as part of the telehealth service are distinct from general practice and have the authority to provide clinical care, for example by adjusting treatment and/or reassuring patients. In some instances, the patient contact may go directly to the hospital consultant [4]. Conversely, telehealth might increase the need for support from primary care, if the extra clinical information obtained through telehealth prompts calls for intervention from professionals. This could be due to abnormal readings that would in the absence of monitoring have returned to a normal range; or due to heightened awareness from patients.

Few studies have addressed the impact of telehealth on primary care. Some small studies have observed indications of time savings for general practices [5,6] and one noted a non significant reduction in home nursing visits and a reduction in hospital admission [7]. A randomised trial of 40 patients’ with moderate to severe Chronic Obstructive Pulmonary Disease (COPD) found that telemonitoring did not change the rate of emergency hospital visits but did reduce primary care contacts for chest problems [8]. A more recent randomised trial of remote blood pressure monitoring in 401 patients with hypertension found improved control of blood pressure but increased general practitioner and nurse consultations [9,10]. The Whole Systems Demonstrator programme aimed to address weaknesses in the evidence base for the effectiveness of telehealth in people with COPD, heart failure and diabetes, through a wide-ranging evaluation in three sites in England, UK (Cornwall, Kent and Newham) [11]. Over 3,000 patients were recruited and received telehealth or usual care on the basis of randomised allocations made at the general practice level. The first published results showed fewer urgent and unplanned (‘emergency’) hospital admissions among telehealth patients than usual-care controls [12], though this appeared to have been linked with increases in emergency admissions among the control group following the start of the trial. Overall savings through reduced hospital activity over one year were not statistically significant. Though reduced mortality rates were seen, there was no overall improvements in quality of life among survivors [13], and the intervention was not found to be cost effective at realistic willingness-to-pay thresholds [2].

In this paper, we describe secondary analysis of the General Practice (GP) data sets amassed for the Whole Systems Demonstrator evaluation. The focus here is on the impact of telehealth on primary care physicians (general practitioners) and on nurses based in general practices (practice nurses). To assess this we compared changes between intervention and control groups in terms of the frequency of visits to general practitioners and practice nurses. We also addressed the number of times clinical readings relevant to the management of patients were recorded on the general practice systems.

## Methods

The protocol for the Whole Systems Demonstrator evaluation has previously been described and included plans to undertake secondary analysis of primary care use [11,12]. The study randomised general practices to offer either telehealth or usual care to eligible patients (criteria in Table 1) with COPD, diabetes or heart failure. Control patients received usual care and were offered telehealth at the end of the twelve-month trial period if they were still eligible at that point.

**Table 1:** Eligibility for the trial

	
Practice characteristics	All practices within the geographical areas covered by the sites (Cornwall, Newham and Kent) were eligible and were invited to participate in the trial by letter.
	Each practice that accepted the invitation to participate was allocated to an intervention or control group via a centrally-administered minimisation algorithm that aimed to ensure that the groups of practices were similar in terms of practice size, deprivation index, proportion of non-white patients, prevalence of diabetes, COPD and heart failure, and site (Cornwall, Kent and Newham).
Patient characteristics	Within each practice, patients aged 18 or over were deemed eligible on the basis of a diagnosis in primary or secondary care for COPD, heart failure or diabetes.
	Eligibility was not conferred on the basis of formal clinical assessment of disease severity. Instead patients were deemed eligible on the basis of either (i) their inclusion on the relevant Quality Outcomes Framework register in primary care, (ii) a confirmed medical diagnosis in primary or secondary care medical records as indicated by general practice Read Codes or ICD-10 codes, or (iii) confirmation of disease status by a local clinician (i.e. general practitioner or community matron) or by their hospital consultant.
	Patients were not excluded on the basis of additional physical co-morbidities. However, the patient’s home had to be suitable for the installation of telehealth.

### Telehealth intervention

The pragmatic trial design meant that there was flexibility for local teams to develop their own telehealth services. Therefore, choices of telehealth devices and monitoring systems varied between the three trial sites and there was no attempt to standardise across sites. Sites used different protocols for allocating peripheral devices but across all sites the critical devices per condition were pulse oximeters (for COPD), blood glucose monitors (for diabetes) and weighing scales (for heart failure). In addition, almost all intervention participants received blood pressure monitors. Participants were asked to take clinical readings up to five days per week at the same time each day. In addition symptom questions and educational messages were transmitted to participants, either via the telehealth base unit or using a set-top box connected to a television. At the end of each session, data were transmitted to monitoring centres via secure servers. These centres were staffed by local health organisations. Sites used a variety of approaches to respond to triggers generated by worsening patterns in telehealth readings. These ranged from an approach based on locating specialist nurses in monitoring centres to routing alerts to community-based staff, such as community matrons. In one of the two Kent Primary Care Trusts, warning signs from telehealth were routed direct to general practices for their response.

### Data sets

Information about service activity was derived from extracts from operational information systems and in particular exploiting the system of coded data on computerised general practice systems. Though there are national standard coding systems available and widely used, there can be differences between practices in the specify codes used. All general practices participating in the trial were asked to share data for the whole of their adult practice population, including registration and encounter dates, diagnoses, test results and prescriptions. Data were “pseudonymised” before being transferred to the research team, so that patient-identifiable fields could be removed and a unique patient identifier (the “NHS number”) encrypted. Pseudonymised NHS numbers were used to link the general practice data to hospital data sets which included information on inpatient stays, outpatient attendances and emergency visits. The research team created person-level data sets to summarise all key health care contacts experienced by the participants over several years. The approach of using pseudonyms in secure environments is regarded by the National Information Governance Board as appropriate technique for these types of study where patient consent across a whole population is not feasible. The study was approved by Liverpool Research Ethics Committee (ref: 08/H1005/4).

### Study cohort and end points

This study was restricted to participants (linked to GP data) that were enrolled into the trial before the planned recruitment termination date (30 September 2009). The trial enrolment date was taken as the date of telehealth installation for intervention patients, and as the date of the initial project team visit for controls. Further, we required that patients had a continuous prior record of registration with general practices within the data sets, spanning the two years prior to the trial and the period of the trial itself (excluding patients that died).

Analysis was based on comparing activity over 12 months from the enrolment dates, at the person level. General practice workload was based on the number of contacts with a principal general practitioner, locum or registrar (coded as staff types A, B or C); and practice nurses in primary care (staff type D06). In both cases, contacts were obtained from the computerised practice systems and included all locations (GP Surgery, Home Visit, Telephone etc.). We also studied the number of times test results were recorded within the general practice records specifically for glycosylated haemoglobin A1C, weight, blood oxygen levels and respiratory flow (relevant Read codes in Additional file 1). These metrics were chosen as they were akin to the readings that were regularly made by patients receiving telemonitoring. A variable was created based on the count of different readings obtained per person.

### Statistical analysis

We examined the similarity of intervention and control patients at baseline using the standardised difference, defined as the difference in sample means as a proportion of the pooled standard deviation [14]. Previous studies have used a standardised difference of 10% as a threshold to denote meaningful difference in baseline variables [15].

To provide information on the generalisability of trial results, we tested for differences between the general practice contact rates of trial participants and those of the wider adult population (among the practices that provided data for the evaluation). In this analysis, these comparisons were based on the number of contacts in 2009/10 and were adjusted for age band and sex using Poisson regression. As not all patients were alive and registered with a practice for the whole of the 2009/10 year, we annualised rates by including an offset in the Poisson regression.

Individuals were analysed on an “intention-to-treat” basis, i.e. based on the randomised treatment allocations of the general practices, and regardless of subsequent withdrawal from the trial. We used two methods to compare intervention and control groups. The first used a difference-in-difference method to assess whether the number of contacts and recorded clinical readings increased more quickly or more slowly among the intervention group than controls, from the year before enrolment to the year after. This was done using ordinary least squares regression, with the output relating to an absolute number of admissions. The second analysis made a direct comparison of numbers of contacts and clinical readings experienced within the twelve-month trial period. This was done using Poisson regression, with exponentiation of the model coefficients producing an incidence rate ratio. As in the primary analysis [12], three versions of the incidence rate ratio were produced, with different forms of case-mix adjustment (Table 2).

**Table 2:** Three forms of case-mix adjustment used in analysis

	
Unadjusted	The simplest models, although accounting for the effect of clustering, used no additional case-mix adjustment.
Adjusted	These models additionally controlled for residual imbalances in a set of baseline characteristics. This set included age, sex, ethnicity, site, number of chronic health conditions, principal long-term condition (diabetes, chronic obstructive pulmonary disease or heart failure), an area-based socioeconomic deprivation score (national quartiles of the Index of Multiple Deprivation 2007), and a metric corresponding to the endpoint (*e.g.*, general practitioners contacts) calculated over several periods within the two years prior to recruitment.
	The number of chronic health conditions was a count of diagnoses recorded on inpatient data over the three years prior to starting the trial. Principal long-term conditions were assigned using a pragmatic approach according to published criteria [16].
Combined model	More complex case-mix adjustment was conducted using the Combined Predictive Model [16] a standard instrument designed to estimate the probability that an individual will experience an emergency hospital admission in a future twelve month period. The Combined Model score uses 72 variables covering age, sex, recorded health conditions, prior hospital use and prescribing, but not primary care contacts. These variables are sourced from general practice and hospital administrative data. Where a general practice did not grant approval to extract data for the evaluation, or where scores could not be calculated, scores were imputed for its patients based on the available information, which included age, sex and the hospital variables. Single imputation was used based on linear regression on the logit scale. When used in the case-mix adjustment, the Combined Predictive Model score was calculated for each participant at the end of the month prior to the start date.

We expected that contact rates would be more highly correlated for patients registered at the same practice than at different practices. Therefore, all of our models reflected intracluster correlation at the practice level, using multilevel models with random effects at the practice level [17].

## Results

### Patient recruitment

249 out of 254 practices agreed to share routine data from their systems for the evaluation, although data extraction was not possible for a further 5 practices for technical reasons linked with the clinical operating systems used. The data sets obtained spanned more than four years (April 2006 to September 2010) and contained almost one billion records. Ultimately, 1,625 control and 1,605 intervention patients were recruited into the telehealth part of the trial from 179 general practices (for full Consort flow diagram see Steventon et al. [12]).

The analysis of general practice use was restricted to 1,219 intervention and 1,098 control participants recruited before September 2009 and with a continuous general practice registration (76% of intervention patients and 68% of controls). Excluded participants were not statistically different from those included in terms of age at baseline, sex and number of chronic conditions. However, included participants were more likely to come from Newham (31.0% of included patients compared with 26.7% of the overall sample), and from the 20% most socioeconomically deprived areas (21.4% of patients compared with 19.5%). Of patients included, a full Combined Predictive Model score could be computed for 2,238 patients (96.6%); scores were imputed for the remainder using the method described in Table 2.

The routine data sets showed that, among the general population in 2009/10, the consultation rates for trial participants were higher than the general population (incidence rate ratio 2.00, p < 0.001) and showed no clear trend by age. Similar patterns were found for practice nurse contacts (incidence rate ratio 1.97, p < 0.001).

### Baseline differences

Intervention and control patients were similar at baseline (Table 3), with only a few standardised differences greater than 10%. The largest standardised differences related to the proportion of people living in the most socioeconomically deprived fifth of the population (8.7% intervention and 5.1% of controls, standardised difference 14.3%) and to long-term health conditions (27.1% of intervention patients had diabetes as their principal condition compared with 22.6% of controls, standardised difference 10.4%).

**Table 3:** Baseline characteristics of intervention and controls groups (data are % of group unless otherwise specified)

	**Control**	**Intervention**	**Standardised difference (%)**
Number in group	1098	1219	
Number of practices	80	82	
Number of patients per practice (median (range))	10 (1 to 62)	8 (1 to 76)	
Index long-term condition			
Chronic Obstructive Pulmonary Disease	47.4	45.0	−4.8
Diabetes	22.6	27.1	10.4
Heart failure	30.0	27.9	−4.6
Number of chronic health conditions (mean (SD))	1.9 (1.8)	1.8 (1.8)	−3.9
Site			
Cornwall	32.6	36.5	8.2
Kent	36.2	32.7	−7.2
Newham	31.2	30.8	−1.0
Age (mean (SD))	70.8 (11.8)	69.7 (11.6)	−9.3
Aged under 65	28.7	30.1	3.1
Aged 65-74	31.4	34.9	7.5
Aged 75-84	30.9	27.4	−7.7
Aged 85+	9.0	7.5	−5.3
Female (%)	40.4	41.1	1.3
Ethnicity			
White	71.3	71.8	1.0
Non-white	13.2	12.3	−2.7
Unknown	15.5	15.9	1.2
Area-level deprivation (mean (SD))*	29.8 (13.8)	28.8 (14.9)	−6.9
1^st^ quartile	5.1	8.7	14.3
2^nd^ quartile	15.4	15.5	0.2
3^rd^ quartile	31.8	32.6	1.9
4^th^ quartile	47.7	43.2	−9.2
GP visits per person (prior year) (mean (SD))	9.0 (7.6)	8.8 (6.8)	−2.0
None	4.5	3.5	−4.8
1-5	35.2	33.1	−4.6
6-10	29.0	30.5	3.4
11-20	23.2	26.4	7.4
>20	8.1	6.5	−6.3
Practice nurse contacts per person (prior year) (mean (SD))	6.1 (8.1)	5.3 (7.8)	−10.2
None	14.8	15.5	2.1
1-5	51.1	57.1	12.1
6-10	16.4	14.8	−4.5
11-20	11.9	7.8	−13.9
>20	5.8	4.8	−4.4
Combined Model score (mean (SD))**	27.0 (20.2)	26.1 (20.1)	−4.3
Low risk	14.6	15.9	3.5
Moderate risk	30.0	32.1	4.5
High risk	44.3	41.6	−5.6
Very high risk	11.1	10.5	−1.9

SD = Standard deviation.

*n(controls) = 1096, n(intervention = 1214). First quartile is least deprived, fourth quartile is most deprived.

**n(controls) = 1,047; n(intervention) = 1,191. Risk categories denote top proportions of site population: very high risk (0.5%), high risk (0.5-5%), moderate risk (5-20%), and low risk (20-100%).

Before the start of the trial, rates of general practitioner contact were similar for the intervention and control groups, at around one visit every 6 weeks (8.8 vs. 9.0 visits per person per year, standardised difference 2.0%). Some patients (constituting 8.1% of control patients) had over 20 visits with a general practitioner during the year before the start of the trial, while 4.5% had no recorded contacts on general practice information systems. The average number of practice nurse contacts during the year before enrolment was slightly lower in the intervention than control group (5.3 contacts per person per year versus 6.1, standardised difference 10.2%). Over both groups 15.2% of patents had no recorded practice nurse contacts whilst 5.3% of participants had over 20 contacts in the year before the start of the trial.

**Figure 1:**
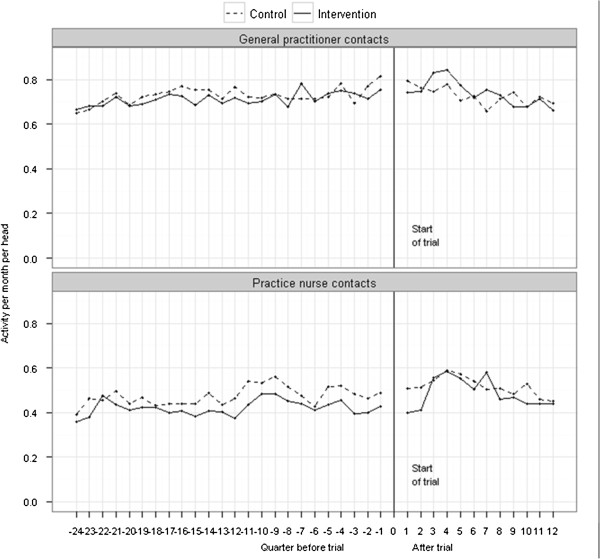
Crude monthly rates of contact.

Figure 1 shows trends in general practitioner and practice nurse contacts by month, without adjusting for baseline covariates. The numbers of visits per person were stable before the start of the trial. During the 12 months of the trial, the number of visits fell slightly for the whole cohort. However, a number of patents died during the 12 months (4.9% of intervention patients included in this sample, compared with 9.1% of controls in the sample). The average quarterly rate of general practitioner contacts for survivors was 2.14 for intervention patients at the end of the trial and 2.26 for controls. Mortality may therefore explain some of the slight fall in visits.

### Analyses of differences in contact rates during the trial period

As previously stated, the difference-in-difference analysis and Poisson regressions took clustering into account; the intracluster correlation coefficient (ICC) for general practitioner contacts was estimated to be 0.184.

During the 12 month trial period, the number of general practitioner contacts in the intervention group (mean 8.99, standard deviation 7.00) was similar to that in the control group (mean 8.85, standard deviation 8.16), see Table 4. When compared with the contacts during the 12 months before the trial started, the difference-in-difference estimate indicated that contacts rose more quickly among the intervention than control group, by 0.29 contacts per head. However, this was not statistically significant (p=0.465) with an adjusted incidence rate ratio was 1.04 (95% confidence interval, 0.95 to 1.30, Table 5). Differences did not reach statistical significance after case-mix adjustment using Poisson regression. While the estimates for rise in contacts were higher for practice nurses than for general practitioners (difference-in-difference estimate 0.45 contacts per head; adjusted incidence rate ratio 1.04, 95% CI 0.82 to 1.30), they were not statistically significant.

**Table 4:** Unadjusted rates of contacts and readings (figures are numbers per patient over twelve months (SD))

	**Before**		**After**		**Difference-in-difference**	
	**Control**	**Intervention**	**Control**	**Intervention**	**Estimate**	**P**
GP contacts	8.98 (7.61)	8.84 (6.76)	8.85 (8.16)	8.99 (7.00)	0.29	0.465
Practice nurse contacts	6.07 (8.07)	5.26 (7.76)	6.28 (8.98)	5.92 (9.83)	0.45	0.245
Clinical readings*	2.76 (2.59)	2.73 (2.47)	2.71 (2.53)	2.80 (2.76)	0.11	0.414

*based on number of recordings of HbA1c, weight, blood oxygen, respiratory flow.

**Table 5:** Results of mixed models (data show incidence rate ratio)

**Endpoint (interpretation)**	**Model**	**Estimate (95% CI)**	**P**
General practitioner contacts	Unadjusted	1.05 (0.90 to 1.23)	0.520
	Adjusted	1.04 (0.95 to 1.14)	0.404
	Combined model	1.04 (0.90 to 1.21)	0.560
Practice nurse contacts	Unadjusted	1.14 (0.81 to 1.61)	0.438
	Adjusted	1.04 (0.82 to 1.30)	0.756
	Combined model	1.13 (0.81 to 1.58)	0.468
Clinical readings*	Unadjusted	1.01 (0.85 to 1.20)	0.881
	Adjusted	1.00 (0.90 to 1.12)	0.931

*based on number of recordings of HbA1c, weight, blood oxygen, respiratory flow.

The number of clinical readings summed across the four clinical domains was similar between intervention and control groups in the 12 months before the trial (2.73 versus 2.67 readings per person). The difference-in-difference estimate indicated that the number of readings increased more quickly among the intervention than control group (by 0.11 readings per head) but this was not statistically significant (p=0.414). The adjusted Poisson analysis reported very similar rates of clinical readings during the twelve months of the trial (adjusted incidence rate ratio 1.00, 95% CI, 0.90 to 1.12, p=0.931).

## Discussion

### Summary

This study addressed a weakness in the evidence base about the impact of telehealth on primary care by exploiting operational data sets from 179 general practices. On average, over the twelve months before the start of the Whole Systems Demonstrator trial, participants had 8–9 consultations with general practitioners, which was substantially higher than for the general population of the three sites during 2009/10 and more consultations than reported in other studies for people of a similar age [18].

We found no evidence to support the theory that telehealth alters rates of contact with general practices. Our adjusted estimate suggested telehealth was associated with an increase of 4% in general practitioner consultations but this was not statistically significant on this sample. We also found no changes in the frequency with which clinical measurements such as weight, HbA1c, blood oxygen and respiratory flow were recorded on the general practice records. Given the frequency with which patients themselves recorded similar measurements using telehealth equipment (up to five times per week), our findings may indicate scope to improve the integration of information systems and care between the telehealth intervention and general practice.

### Strengths and limitations

This particular study is part of a wider evaluation of the Whole Systems Demonstrator programme, which addresses a wider range of outcomes than in this paper. We hypothesised that telehealth could act in two opposing ways: either to increase or reduce the need for routine contact - though we found no overall effect it may be these two effects occurred but offset each other. Alternatively, it is possible that the impact of telehealth on workload was not manifested simply in the numbers of consultations but may be in the nature of consultations. For example, the content of general practice encounters might have changed following introduction of telehealth, even if the number of overall contacts did not change.

This study (with 2,317 patients included in the analysis) was much larger than the previous hypertension trial (which recruited 401 patients) [9]. However, it is possible that issues of sample size have contributed to the lack of statistical significance of the findings; we note we cannot rule out changes as great as a 10% reduction in general practitioner contacts or a 23% increase.

We relied on pre-existing, routine data sets. This has benefits over the alternative approach using self-reported data from patients, as patients do not always recall the use of health services accurately, particularly when it comes to events with low ‘salience’ including many general practice visits [19]. However, using existing administrative or clinical data sets may also bring problems with the completeness of data [20,21]. For example, problems with the completeness of coding meant that we were not able to separate surgery consultations from home visits. Similarly it may be that some tests may have stayed as written comments in the notes, and not been coded on the computer systems.

In a cluster-randomised trial there is a risk of selection bias. Although patients were blinded to treatment allocations until after they had consented to participate, it was not possible in this trial to guarantee that those people recruiting patients were always blinded. Therefore, there is a risk that patients with certain characteristics might be preferentially recruited into either the control or the intervention arm. We were reassured that we could observe few large differences between the characteristics of intervention and control patients at baseline, though as previously noted the number of participants per practice differed for intervention and control practices (medians 8.5 and 12). We were able to adjust for observed baseline characteristics using several forms of case-mix adjustment, and also applied a difference-in-difference estimator.

The study aimed to address a broad class of telehealth devices and did not aim to address specific devices and monitoring systems. Therefore these findings reflect a routine introduction of telehealth, which differed between sites based on a series of decisions by local teams. Thus, the monitoring systems used by the sites involved general practice staff to varying extents. Although this plurality may be seen as problematic to those wanting to replicate specific aspects of the interventions, in some ways it is the merit of a pragmatic trial as it meant we could reflect implementation decisions made by local teams.

The large, multicentre nature of this trial should improve the generalisability of the trial results. However, the patients and health care professionals who agree to participate in randomised controlled trials may differ from those who would use telehealth in routine clinical practice [22]. In this study, the participants were higher users of general practice services than other adult patients, but this was expected given that eligible patients had a long-term condition. A qualitative study that interviewed eligible patients who refused to participate in this trial found three broad reasons, including issues relating to the technology, self-care plans and perceived threats to existing services [23]. Also of interest is whether either of the treatments received in this trial (telehealth or control) might have differed from those offered in routine clinical practice. We note that staff in the three trial sites reported that the randomised nature of the trial constrained them, so that they were not able to innovate and improve the design of the telehealth trial in response to learning [24]. Further, telehealth devices are changing, and may have different impacts according to the nature of the surrounding services and context. Thus, the impact of telehealth in other settings might differ from that reported in this study.

The initial analysis of hospitalisation data found that emergency hospital admission rates appeared to increase for the control group following enrolment [12]. This leads us to suggest that either the trial protocol changed the management of patients allocated to this group, or that control patients may have reacted to their allocations, leading them to seek more care at the accident and emergency department. We note that, in the current study, we did not see a corresponding increase in general practice visits.

### Comparison with existing literature

Most studies of telehealth that address impacts on service use focus on hospital activity [25]. Up until recently, studies that looked at primary care tended to focus on specific populations or issues, such as mental health problems or geographically-dispersed communities. The few studies that have considered similar forms of telehealth to those tested in this trial, have often reported more positive findings than here. For example, a feasibility study in 20 elderly patients indicated a time saving for the GP [6], while a study of home telemonitoring in heart failure patients found reductions in use of clinic visits [26]. However these studies were much smaller than this trial and for this reason may have recruited unrepresentative patients. Wade and colleagues commented on how telehealth alerts prompted frequent contacts and increased the case managers’ workload [27]. A recent randomised study of telehealth in hypertension found approximately one additional GP surgery consultation and half a practice nurse surgery consultation per person in the intervention group compared with the usual care group [9], though in that study general practice teams were more directly involved with the telemonitoring than in the current study.

### Implications

This study used linked operational data for large numbers of patients, a method that has considerable potential for future studies especially those that use retrospective analyses. The lack of observed impacts of telehealth suggests that fears about increases in general practice workload may be unfounded, at least when it comes to workload for clinical staff as measured by the number of contacts. The absence of any statistically-significant effects will not by itself provide a financial or workload incentive for primary care to deliver telehealth. However, under recent changes to the NHS, clinical commissioning groups including primary care professionals may have a firmer financial incentive to prevent hospital admissions.

Telehealth may have different impacts on general practice workload if designed differently. For example, we note that many of the measures captured by telehealth are recorded by general practices (such as weight and blood oxygen levels). In theory, this means there is potential for some work to be shifted from clinical staff to patients. However, we did not find that these readings were recorded in general practices any less frequently among the intervention group. Better information systems and higher general practice engagement might lead to more impressive changes. We note however, that clinical guidelines require some of these readings to be recorded in general practice and remuneration systems track the number of such measurements that are made. For diabetes, HbA1c is recognised as a more stable measure than blood glucose. This illustrates how the consequences of new technology depend on the wider health systems, and not only on the attributes of the technology itself.

## Conclusions

In this study, the use of home based telehealth for people with chronic disease did not appear associated with changes in the frequency with which people contacted general practitioners and practice nurses. This suggest that fears that the widespread increase in the use of this technology may increases the burden on primary are unfounded. Conversely, we did not find evidence that telehealth led to a significant reduction in GP workload. We note that the way that telehealth impacts on primary care roles may be influenced by a number of other features in the health system. The challenge is to ensure that these systems lead to better integration of care than fragmentation.

## Competing interests

All authors have completed the Unified Competing Interest form at http://www.icmje.org/coi_instructions.html (available on request from the corresponding author) and declare: no support from any organisation for the submitted work besides that described from the Department of Health; no financial relationships with any organisations that might have an interest in the submitted work in the previous 3 years; no other relationships or activities that could appear to have influenced the submitted work. A number of the authors undertake evaluative work funded by Government or public agencies but these do not create competing interests.

## Authors’ contributions

MB designed this element of the work, contributed to the analysis and wrote the first draft of the paper. AS contributed towards the design of the work, led on the collection of administrative data sets and conducted the analysis. HD contributed statistical advice. All authors revised the manuscript. All authors read and approved the final manuscript.

## Pre-publication history

The pre-publication history for this paper can be accessed here:

http://www.biomedcentral.com/1472-6963/13/395/prepub

## Supplementary Material

Additional file 1 Read codes used to define clinical readings. Click here for file

## References

[B1] Department of Health Whole system demonstrator programme: headline findings 2011 Available at: http://www.dh.gov.uk/prod_consum_dh/groups/dh_digitalassets/documents/digitalasset/dh_131689.pdf [accessed 2012 07 23]

[B2] Henderson C Knapp M Fernandez J-L Beecham J Hirani SP Cartwright M Rixon L Cost effectiveness of telehealth for patients with long term conditions (whole systems demonstrator telehealth questionnaire study): nested economic evaluation in a pragmatic, cluster randomised controlled trial BMJ 2013 346 mar20 4 f1035 doi:10.1136/bmj.f1035 10.1136/bmj.f1035 23520339

[B3] May CR Finch TL Cornford J Exley C Gately C Kirk S Jenkings KN Osbourne J Robinson AL Rogers A Wilson R Mair FS Integrating telecare for chronic disease management in the community: what needs to be done? BMC Health Serv Res 2011 11 131 10.1186/1472-6963-11-131 21619596 PMC3116473

[B4] Mclean S Protti D Sheik A Telehealthcare for long term conditions BMJ 2011 342 d120 doi:10.1136/bmj.d120 10.1136/bmj.d120 21292710

[B5] Terschüren C Fendrich K van den Berg N Hoffmann W Implementing telemonitoring in the daily routine of a GP practice in a rural setting in northern Germany J Telemed Telecare 2007 13 4 197 201 10.1258/135763307780908003 17565776

[B6] Roberts A Garrett Lm Godeen DJ Can telehealth deliver for rural Scotland? Lessons from the Argyll & Bute telehealth programme Scott Med J 2012 57 33 37 doi:10.1258/smj.2011.011288 10.1258/smj.2011.011288 22408213

[B7] Martín-Lesende I Orruño E Bilbao A Vergara I Cairo1 C Bayón JC Reviriego E Romo MI Larrañaga J Asua J Abad R Recalde E Impact of telemonitoring home care patients with heart failure or chronic lung disease from primary care on healthcare resource use (the TELBIL study randomised controlled trial) BMC Health Serv Res 2013 13 118 10.1186/1472-6963-13-118 23537332 PMC3636109

[B8] Lewis KE Annandale JA Warm DL Rees SE Hurlin C Blyth H Syed Y Lewis L Does home telemoniroting after pulmonary rehabilitation reduce healthcare use in optimized COPD? A pilot randomised trial J Chronic Obstructive Pulm Dis 2010 7 44 50 10.3109/15412550903499555 20214462

[B9] McKinstry B Hanley J Wild S Pagliari C Paterson M Lewis S Sheikh A Krishan A Stoddart A Padfield P Telemonitoring based service redesign for the management of uncontrolled hypertension: multicentre randomised controlled trial BMJ 2013 346 f3030 doi:10.1136/bmj.f3030 (Published 24 May 2013) 10.1136/bmj.f3030 23709583 PMC3663293

[B10] Stoddart A Hanley J Wild S Pagliari C Paterson M Lewis S Sheikh A Krishan A Padfield P McKinstry B Telemonitoring-based service redesign for the management of uncontrolled hypertension (HITS): cost and cost-effectiveness analysis of a randomised controlled trial BMJ Open 2013 3 5 doi:10.1136/bmjopen-2013-002681 10.1136/bmjopen-2013-002681 PMC3657667 23793650

[B11] Bower P Cartwright M Hirani SP Barlow J Hendy J Knapp M A comprehensive evaluation of the impact of telemonitoring in patients with long-term conditions and social care needs: protocol for the whole system demonstrator cluster randomised trial BMC Health Serv Res 2011 11 184 10.1186/1472-6963-11-184 21819569 PMC3169462

[B12] Steventon A Bardsley M Billings J Dixon J Doll H Hirani S Cartwright M Rixon L Knapp M Henderson C Rogers A Fitzpatrick R Hendy J Newman S Effect of telehealth on use of secondary care and mortality: findings from the whole system demonstrator cluster randomised trial BMJ 2012 344 e3874 10.1136/bmj.e3874 22723612 PMC3381047

[B13] Cartwright M Hirani SP Rixon L Beynon M Doll H Bower P Bardsley M Effect of telehealth on quality of life and psychological outcomes over 12 months (whole systems demonstrator telehealth questionnaire study): nested study of patient reported outcomes in a pragmatic, cluster randomised controlled trial BMJ 2013 346 feb26 2 f653 f653 doi:10.1136/bmj.f653 10.1136/bmj.f653 23444424 PMC3582704

[B14] Flury BK Reidwyl H Standard distance in univariate and multivariate analysis Am Stat 1986 40 249 251

[B15] Normand ST Landrum MB Guadagnoli E Ayanian JZ Ryan TJ Cleary PD Validating recommendations for coronary angiography following acute myocardial infarction in the elderly: a matched analysis using propensity scores J Clin Epidemiol 2001 54 4 387 398 10.1016/S0895-4356(00)00321-8 11297888

[B16] Wennberg D Siegel M Darin B Combined predictive model: final report and technical documentation 2006 London: Kings Fund Available at: http://www.kingsfund.org.uk/sites/files/kf/field/field_document/PARR-combined-predictive-model-final-report-dec06.pdf

[B17] Campbell MK Grimshaw JM Elbourne GR Intracluster correlation coefficients in cluster randomized trials: empirical insights into how they should be reported BMC Med Res Methodol 2004 4 doi:10.1186/1471-2288-4-9 10.1186/1471-2288-4-9 PMC415547 15115554

[B18] Hippisley-Cox J Vinogradova Y Trends in consultation rates in general practice 1995 to 2008:analysis of the QResearch® database. Final report to the NHS Information Centre and Department of Health 2009 Leeds: The Health and Social Care Information Centre

[B19] Bellón J a Lardelli P Luna JD Delgado a Validity of self reported utilisation of primary health care services in an urban population in Spain J Epidemiol Community Health 2000 54 7 544 551 10.1136/jech.54.7.544 10846198 PMC1731703

[B20] Lezzoni LI Assessing quality using administrative data Ann Intern Med 1997 127 666 674 10.7326/0003-4819-127-8_Part_2-199710151-00048 9382378

[B21] Roos LL Mustard CA Nicol JP McLerran DF Malenka DJ Young TK Cohen MM Registries and administrative data: organization and accuracy Med Care 1993 31 201 212 10.1097/00005650-199303000-00002 8450678

[B22] Rothwell PM External validity of randomised controlled trials: “to whom do the results of this trial apply?” Lancet 2005 365 82 93 10.1016/S0140-6736(04)17670-8 15639683

[B23] Sanders C Rogers A Bowen R Bower P Hirani S Cartwright M Exploring barriers to participation and adoption of telehealth and telecare within the whole system demonstrator trial: a qualitative study BMC Health Serv Res 2012 12 220 10.1186/1472-6963-12-220 22834978 PMC3413558

[B24] Hendy J Chrysanthaki T Barlow J Knapp M Rogers A Sanders C An organisational analysis of the implementation of telecare and telehealth: the whole systems demonstrator BMC Health Serv Res 2012 12 1 403 10.1186/1472-6963-12-403 23153014 PMC3532839

[B25] Clark RA Inglis SC McAlister FA Cleland JGF Stewart S Telemonitoring or structured telephone support programmes for patients with chronic heart failure: systematic review and meta-analysis BMJ 2007 334 942 10.1136/bmj.39156.536968.55 17426062 PMC1865411

[B26] Dar O Riley J Chapman C Dubrey SW Morris S Rosen SD Roughton M Cowie MR A randomized trial of home telemonitoring in a typical elderly heart failure population in north west London: results of the home-HF study Eur J Heart Fail 2009 11 3 319 325 Epub 2009 Jan 27 10.1093/eurjhf/hfn050 19174529 PMC2645059

[B27] Wade MJ Desai AS Spettell CM Snyder AD McGowan-Stackewicz V Kummer PJ Maccoy MC Krakauer RS Telemonitoring with case management for seniors with heart failure Am J Manag Care 2011 17 3 e71 79 21504262

