# Supplementary material for: Impact of telehealth on general practice contacts: findings from the whole systems demonstrator cluster randomised trial
Source: BMC Health Serv Res. 2013 Oct 8;13:395. doi: 10.1186/1472-6963-13-395 (PMC3852608; doi:10.1186/1472-6963-13-395)
Supplement: Additional file 1 — Read codes used to define clinical readings. [file 1472-6963-13-395-S1.docx]

**Appendix: Read codes used to define clinical readings**

|  | Version 2 Read Code |
| --- | --- |
| HbA1c | 42W..%, 44TB. |
| Weight | 22K..%, 22A..%, 4I5.., 6878., 66C.., 67I9., R031. |
| Blood oxygen | 44Y40, 44YE., 44Y41, 44YC., 44Y9., 44Y5., 44YA. |
| Respiratory flow | 339..% |
